# Detection of Nora virus in infected *Drosophila melanogaster* heads without evidence of ubiquitous brain infection by smRNA FISH

**DOI:** 10.1080/19336934.2026.2620887

**Published:** 2026-01-22

**Authors:** Blase Rokusek, Shalie Sklenar, Luke J. Hamilton, Sunayn Cheku, Darby J. Carlson, Kimberly A. Carlson

**Affiliations:** Department of Biology, University of Nebraska at Kearney Kearney, NE, USA

**Keywords:** *Drosophila melanogaster* Nora virus (DmNV), brain dissection, neuroinvasion, small molecule RNA fluorescence *in situ* hybridization (smRNA FISH), confocal microscopy, *Noraviridae*

## Abstract

*Drosophila melanogaster* Nora virus (DmNV), a positive-sense single stranded RNA virus related to picornaviruses. Given its genetic and structural similarity to neurotropic picornaviruses, such as poliovirus, we sought to determine whether DmNV could be found within the head and brain of *D. melanogaster*. RNA was extracted from heads of chronically DmNV-infected stocks, as well as from uninfected controls, and assayed using reverse transcription-polymerase chain reaction (RT-PCR) for DmNV *open reading frame 1* (*ORF1*). The results showed that DmNV genomic material can be isolated from the heads of DmNV-infected *D. melanogaster*, which suggests that the virus reaches the head during the course of infection. To determine whether DmNV infects the brain tissue itself, small-molecule RNA fluorescence *in situ* hybridization (smRNA FISH) experiments on whole brains dissected from DmNV-infected and uninfected *D. melanogaster* were done. The smRNA FISH detection method was validated by identifying DmNV RNA in gut tissue, but there was no evidence of DmNV localization in any brain specimens examined. These findings suggest an alternative explanation for why DmNV may be present in dissected head specimens. Additionally, we highlight the effectiveness of smRNA FISH as a highly specific and accessible method for detecting RNA viruses in Drosophila, offering an alternative to antibody-based or transgenic fluorescence approaches. Together, our results refine the understanding of DmNV tissue tropism and provide methodological insights for future studies using insect RNA viruses.

## Introduction

*Drosophila melanogaster* Nora virus (DmNV) was first described less than two decades ago when it was found to infect both laboratory and wild-caught strains [1]. Structurally, DmNV is an icosahedrally symmetric particle, which is similar to viruses of the order Picornavirales, and more precisely to those of the family Picornaviridae. Yet, despite striking similarities to the family Picornaviridae, it became clear that DmNV might be better classified within a new family of ‘picorna-like’ insect viruses [2,3]. Indeed, a plethora of related insect viruses have been discovered [3–8], bolstering the argument for a new family of insect viruses, which has been officially recognized by the International Committee on Taxonomy of Viruses, known as Noraviridae. Even as Noraviridae has been accepted as a family within the order Picornavirales, related viruses continue to be described [9–11], making characterization of this ever-expanding group of viruses increasingly more important and relevant. Further, the related Picornaviridae family of viruses is of high importance to human health and includes poliovirus, coxsackieviruses, hepatitis A virus and rhinoviruses [12]. Given the genetic similarity to the viruses of this family, DmNV and other insect picorna-like viruses can be valuable models for research.

When DmNV was first discovered and characterized, it was understood to cause primarily asymptomatic infection [1,13]. However, since that time, some measurable effects of DmNV infection have been described [14], including a reduction in number of viable offspring from infected females, increased bacterial load, impaired locomotor function and increased sleep time [15]. Rogers et al. [16] also showed that DmNV negatively impacts locomotor function in *D. melanogaster*, as assessed by a series of geotaxis assays, though the difference was only evident when thousands of infected *D. melanogaster* were assayed. Whether the locomotor effects can be attributed to a specific neuromotor dysfunction versus a general reduction in fitness during infection remains unresolved. One possible explanation is that DmNV directly infiltrates the *D. melanogaster* nervous system, as viruses in the Picornaviridae family do within their respective hosts. For example, Fujiyuki et al. [17] identified the presence of Kakugo virus, a picornavirus, in the brains of worker honeybees, correlating with aggressive behaviour in the host. Similarly, human picornaviruses, such as poliovirus, have long been known to show neurotropism within the human host, correlating with neuromotor signs and symptoms [18,19]. In this context, insect viruses could offer a useful means to study viral neuroinfections in general [20,21], making investigation important.

To our knowledge, no reports of measurable DmNV localization within heads of infected *D. melanogaster* have been published. In fact, the first characterization of DmNV tissue localization found negligible virus within heads by relative expression RT-PCR assays [13]. However, DmNV-specific, small interfering RNAs (siRNA) have been isolated from the heads of DmNV-infected *D. melanogaster*. These 21-nucleotide DmNV RNA sequences could be presumed to represent the duplex RNA products of ribonuclease Dicer-2 in response to dsRNA replication intermediates [22], suggesting the presence of an immune response to DmNV replication within the head. Yet recent data could offer an alternative explanation for the presence of DmNV-specific siRNAs within virus-naive tissue in the context of a viral DNA amplification pathway by endogenous reverse transcription [23–25]. Nevertheless, we found it worthwhile to explore the possibility that DmNV could reach the head of infected organisms and show neurotropism within the brain.

The primary impetus for the present investigation was to further characterize DmNV infection in adult *D. melanogaster*, especially in relation to a potential for head and brain localization. To start, RNA was extracted from dissected heads of infected *D. melanogaster* and the presence of DmNV genetic material within the whole tissue confirmed. To determine whether DmNV identified within the heads of infected *D. melanogaster* is the result of brain tissue infiltration specifically, we dissected whole brains and utilized small-molecule RNA fluorescence *in situ* hybridization (smRNA FISH) and confocal microscopy. We found no evidence for DmNV within the brains of infected organisms in any of the specimens examined. Finally, while validating the use of smRNA FISH to detect DmNV genetic material, we found the nucleic acid identification technique to be an easy and effective alternative compared to immunofluorescence or transgenic fluorescence techniques to detect RNA viruses within *D. melanogaster* tissue.

## Methods

### D. melanogaster husbandry

Witi *Rel*^E23^ *D. melanogaster* (a kind gift from Dan Hultmark from Umeå, Sweden [1]) was used for all experiments. This stock has been maintained in our laboratory, representing a direct lineage from the first stock described as infected with DmNV. Further, the use of *D. melanogaster* with white eyes removes the possibility of red-eye pigment interfering with fluorescent microscopy of brain tissue. The stock was maintained at 25°C on standard cornmeal, molasses and torula yeast medium (Bloomington Drosophila Stock Center classic recipe) with a 12:12 light:dark cycle. Both infected and uninfected Witi *Rel*^E23^ stocks were maintained. DmNV uninfected stocks were established and occasionally renewed by collecting and dechorionating eggs to remove viral particles, as described by Weatherred et al. [26]. Whole-body RNA extraction and RT-PCR using DmNV-specific primers (described in detail below) were used to confirm the presence of DmNV infection.

### Formal DmNV infection protocol

All adults were cleared from DmNV-negative bottles. Five days later, newly eclosed adults were sexed under light ether anaesthesia, and females were moved to fresh food vials at a density of approximately 40 organisms per vial while awaiting infection. Meanwhile, 10 male DmNV-infected *D. melanogaster* were put on fresh, standard food to defaecate for 4 days. The males were removed and the experimental females added to the vials to be exposed to the virus. Uninfected control females of the same age as the experimental organisms were kept on fresh and uninfected food in a separate incubator. Organisms were transferred to vials containing only a cotton ball soaked in 5% sucrose water for 1 day, in an attempt to limit surface contamination with DmNV-laden faeces. In other experiments, we did not include this one-day starvation step.

### Persistently infected organisms

To ensure that our DmNV-probes could detect viral material, adult female flies were collected from persistently infected and uninfected stock bottles (infected and uninfected organisms of same age and less than 10 days from eclosion). These organisms were transferred to vials containing only a cotton ball soaked in 5% sucrose water for 1–4 days to remove residual virus from the guts. However, this starvation step did not appear to affect virus detection within the gut.

### RNA extraction and RT-PCR analysis

Heads and bodies were separated from 10 DmNV-infected and -uninfected control females and stored at −80°C until they were processed by RNA extraction. Total RNA was extracted from 10 heads and corresponding bodies for both the DmNV-infected and -uninfected control females. RNA extraction was performed using TRIzol per manufacturer’s instructions (ThermoFisher Scientific, Waltham, MA). Each sample was quantitated using a NanoDrop^TM^ ONE spectrophotometer (ThermoFisher Scientific) to assess RNA purity (260/280 ≈ 2.0) and concentration. RNA samples were stored at −80°C until they were used for RT-PCR. Samples were analysed for the presence of DmNV using DmNV *ORF1* 55–844 (Forward 5-TGGTAGTACGCAGGTTGTGGGAAA-3; Reverse 5-AAGTCATGCTGGCTTCTCAAC-3) primers and *alpha tubulin at 84B* (Forward 5-CCTCATAGCCGGCAGTTCGAACGT-3; Reverse 5-GAGCTCCCAGCAGGCGTTTCC-3 [27]) primers as an endogenous control. RT-PCR was performed using qScript XLT 1-Step RT-PCR (Quantabio, Beverly, MA) according to manufacturer’s instructions. The positive controls were an RNA extraction that previously tested positive for DmNV. Reactions using 250 ng of total RNA were set-up under the following conditions: 50°C for 30 min, 94°C for 2 min, (94°C for 30 sec, 60°C for 30 sec, 68°C for 1 min) for 30 cycles, 68°C for 5 min and hold at 4°C. The DmNV *ORF1* product was expected to be 790bp and *alpha tubulin* to be 187 bp.

### Dissection and fixation of tissue

Guts were dissected from DmNV-infected and -uninfected controls in cold phosphate-buffered saline (PBS; 10 mM sodium phosphate and 0.15 M NaCl at pH 7.5) as previously described [28]. Whole brains were dissected in cold PBS, as described previously [29–31]. Immediately following dissection, brains and guts were fixed with 4% paraformaldehyde diluted in PBS with 0.3% Triton-X (PBSTX) for 1–2 hours. The fixative was removed, and the specimens were rinsed three times with 500 uL of PBS with 0.3% Tween 20 (PBST), followed by three washes for 15 min each with 500 uL PBST. Following the third wash, the specimens were stored for several hours in PBST at 4°C until samples could be further processed.

### smRNA fluorescence in situ hybridization (smRNA FISH) probe design

smRNA FISH probes targeting DmNV were designed with the Stellaris custom probe design software (Biosearch Technologies, Petaluma, CA; https://www.biosearchtech.com/products/rna-fish), following manufacturer instructions and recommendations. Specifically, we used the DmNV *ORF4* sequence (GenBank; National Library of Medicine, accession no. NC_007919) with the probe design software setting the software to 85 probes, with 1 bp spacing, and masking set at 5. The nucleotide Basic Local Alignment Search Tool (BLASTn) from the National Library of Medicine was used to screen the probes for sequence similarity to *D. melanogaster* mRNA transcripts, so as to avoid off-target binding and background noise. Specifically, we searched our probe sequences using the BLASTn tool, setting the Sequence Read Archive (SRA) as the database and searching for transcripts assembled from select cDNA libraries. This project was conceived with the intent to use the smRNA FISH probes within brain tissue, therefore cDNA libraries generated by Brown et al. [32] from female *D. melanogaster* heads at 1, 4 and 20 days post-eclosion were selected. These SRA accession numbers used are listed in Table S1. As per manufacturer recommendation, we discarded any probe sequences that shared 16 or more base pairs of sequence identity with any mRNA transcript from the aforementioned BLASTn hits. We were left with 29 probes passing the screening criteria, which are listed in Table S1. We elected to use Quasar570 (max absorption 548 nm, max emission 566 nm), as suggested by the manufacturer for sets containing less than 30 probes.

After we began to utilize the probes within dissected guts, we wanted to ensure that our probes would also have a low likelihood of off-target binding outside of brain tissue. We again used the BLASTn tool, but this time we searched the remaining 29 probes against the reference RNA sequences (refseq_rna) with the organism set to *D. melanogaster* (taxid: 7227). The hit table was downloaded for all 29 probes, and the dataset was sorted by percent sequence identity and then length of match in terms of base pairs. Only one probe was found to have a hit of 16 or more base pairs with more than 95% identity. The hit table, as well as the parameters for the BLASTn, can be found in the supplemental materials.

### smRNA FISH analysis

The procedure was modified from that described by Yang et al. [33] for quantifying post-transcriptional regulation in *D. melanogaster* brains using a smRNA FISH technique. However, here we targeted DmNV RNA as opposed to endogenous Drosophila mRNA. The PBST solution, in which the fixed specimens were stored at 4°C, was removed and 500 uL of modified wash Buffer A (Biosearch Technologies Stellaris RNA FISH kit; 1.4 mL wash buffer A solution, 4.9 mL nuclease free water, 0.7 mL formamide) was added and incubated at 37°C for 5–20 min with mild shaking. Wash buffer A was removed and 200 µL of hybridization buffer (9:1 buffer to formamide and probes at 1 µM) was added and incubated at 37°C for 12–14 hours with mild shaking and protection from light. The hybridization buffer was removed and the specimens rinsed with 500 µL wash buffer A three times. Following rinses, the specimens were washed three times for 15–30 min with wash buffer A at 25°C and protected from light. Note that during the second wash, the nuclear stain DAPI (1 mg/mL) at a dilution of 1:1000 was added to the wash buffer. After the third wash, the buffer was removed and the specimens were washed once with 500 µL PBST for 15 min at room temperature, while being protected from light. Following the final wash, the specimens were aspirated from the PBST solution with a 100 µL pipette tip pre-wetted with PBST to prevent the tissue from adhering to the pipette tip [30] and moved to a frosted microscope slide. The specimens were arranged and covered with Vectashield mounting medium (Vector laboratories, Newark, CA). A coverslip was added and sealed with CoverGrip™ Coverslip Sealant (Biotium, Fremont, CA).

### Confocal microscopy

Mounted specimens were imaged using a Fluoview3000 confocal laser scanning microscope (Olympus, Tokyo, Japan), fitted with a Galvano scanner, which was used to sequentially scan mounted tissue. A 561 nm laser was used for excitation with the detection wavelength range set to 570–670 nm. A 405 nm laser and a detection wavelength range of 430–470 nm was utilized to detect DAPI. The laser transmissivity and detector gain (PMT Voltage) were kept consistent for all samples imaged within each experiment. Images in z stacks were exported from the confocal microscope as.tif files. An upright Nikon Eclipse T*i* (Tokyo, Japan) inverted microscope fitted with the X-Cite® 120Q (Excelitas Technologies, Singapore) fluorescent illumination box was utilized to facilitate imaging of the whole gut samples. The GFP filter was used to image Quasar570 (smRNA FISH targeting DmNV *ORF4*), while the UV filter was used to image DAPI. The lowest discrete illuminance setting (12%) was used for all images. For all fluorescent images captured, the exposure time was standardized at 400 ms for the GFP filter and 100 ms for the UV filter. Images from the upright fluorescent microscope were exported as.jp2 files.

### Image processing

The biological image analysis plugin for ImageJ, FIJI [34], was used for processing of images. Single channels are presented in grey scale to facilitate contrast perception, and merged images are presented in a cyan/yellow psuedocolor palate. Each single channel image was contrast adjusted to facilitate visibility for publication [35].

For all images taken with the confocal microscope, the auto function within FIJI was used to stretch the contrast histogram for DAPI images, as well as for images with high luminance (i.e. DmNV-infected specimens). Contrast stretch was normalized to the images from infected guts within the DmNV ORF4 channel.

For images of whole guts taken using the inverted fluorescent microscope, a normalized contrast adjustment workflow was utilized as described below. The inverted fluorescent microscope has discrete luminance settings, with the lowest we could employ at 12% of total luminance. To account for noise, a whole gut showing infection and a whole gut not showing infection were each imaged on the confocal microscope using the 20x objective lens. A multi-area-time-lapse function was used to stitch images taken in sequence. The stitched z-stacks (86 slices for the infected gut and 90 slices for the uninfected gut) were summed in FIJI for each specimen for both channels to simulate images captured with a widefield microscope. Some cumulative background noise was appreciated after summing the stacks. To remove this, both summed stacks were auto contrast adjusted for the RNA FISH channel. To standardize the channels for both specimens the minimum and maximum contrast values were adjusted. This effectively removed the background noise in the uninfected gut. After this normalization process, these two specimens functioned as positive and negative controls for the inverted fluorescent microscope.

The same ‘control’ guts were imaged on the inverted fluorescent microscope with the 4x objective lens. On these images, the contrast was manually adjusted in FIJI such that the infected and uninfected guts showed the same pattern of fluorescence for DmNV *ORF4* and DAPI as the respective control images from the confocal microscope. These contrast adjustment parameters were applied to all guts imaged with the inverted fluorescent microscope. The adjusted images representing positive and negative controls can be seen in Figure S1.

## Results

### DmNV nucleic acid is present in heads of infected D. melanogaster

**Figure 1.**
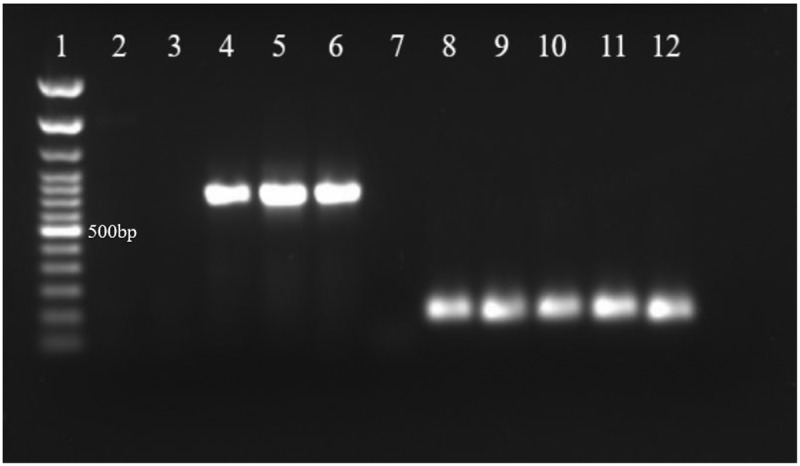
DmNV genetic material detected in heads of DmNV exposed adult *D. melanogaster*. DmNV *ORF1* (790 bp; primers used for reactions in lanes 2–7) and *alpha tubulin* (187 bp; primers used for reactions in lanes 8–12) products were amplified with RT-PCR. A 100bp ladder was loaded into lane 1, followed by RT-PCR products amplified from RNA extracted from heads and corresponding bodies of DmNV-unexposed flies in lanes 2 and 3, respectively. Products from heads and corresponding bodies of exposed flies were loaded into lanes 4 and 5, respectively. Lane 6 (positive control) is the RT-PCR product from RNA previously shown to be DmNV-positive, and lane 7 (negative control) RNA previously shown to be DmNV-negative. Lanes 8 through 10 are *alpha tubulin*-targeted products from heads and corresponding bodies from DmNV-unexposed and -exposed flies, respectively. Lanes 11 and 12 are the *alpha tubulin*-targeted RT-PCR products from RNA previously shown to be DmNV-positive and -negative, respectively.

Ten heads and corresponding bodies were dissected from DmNV-infected and -uninfected *D. melanogaster*. RNA was extracted and RT-PCR was performed with primers targeting DmNV *ORF1*. As an endogenous control, all samples were also used in RT-PCR reactions with primers targeting *alpha tubulin*. A product (~790 bp) correlating with DmNV *ORF1* was amplified from RNA extracted from heads of DmNV-exposed flies (Figure 1).

### smRNA FISH effectively targets DmNV ORF4

**Figure 2.**
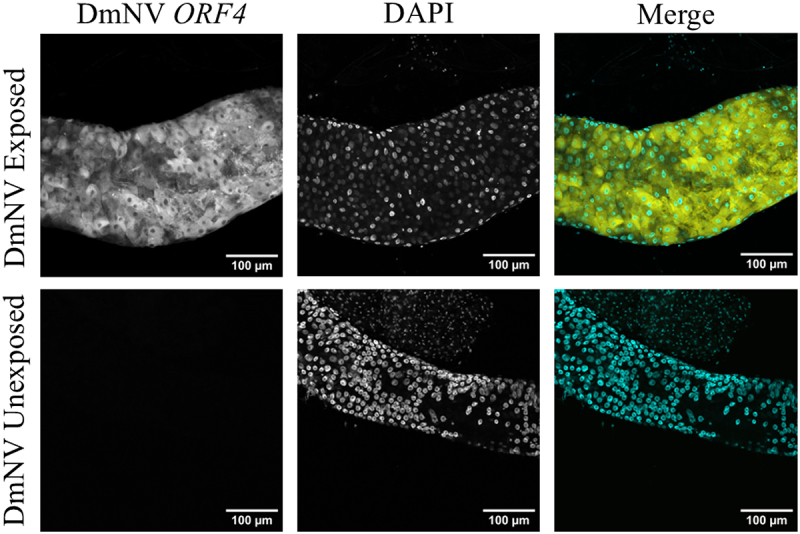
Whole mounted guts from DmNV exposed and unexposed *D. melanogaster*. Images were taken using confocal microscopy. DmNV *ORF4* was targeted by custom-designed smRNA FISH probes. Single channels are presented in greyscale to facilitate contrast perception. In the merged images, DmNV *ORF4* is represented by yellow and DAPI by cyan.

To verify that the smRNA FISH method could be used to image DmNV within tissue, guts were dissected from DmNV-infected and -uninfected *D. melanogaster* in several trial experiments. Confocal microscopy showed strong, unambiguous signals present within the guts of DmNV-infected *D. melanogaster*, with essentially no perceivable noise in the confocal images (Figure 2).

### No evidence for ubiquitous DmNV neurotropism within brain tissue

**Figure 3.**
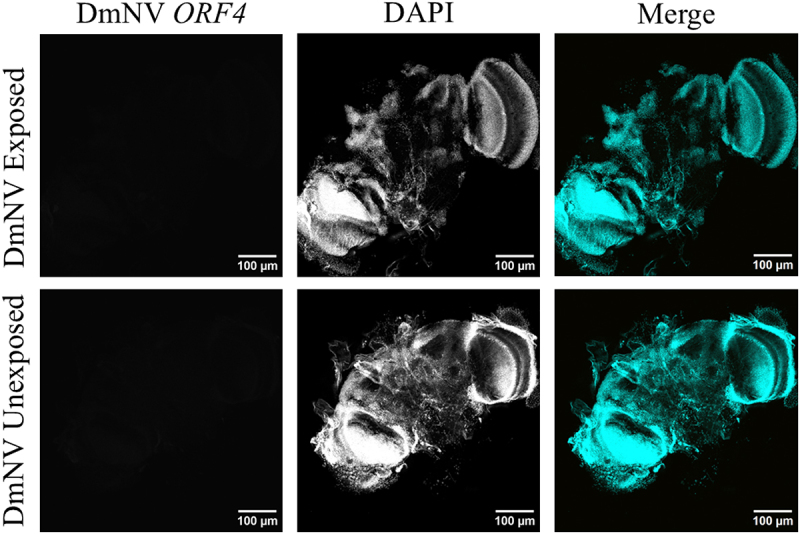
Whole-mounted brains from DmNV exposed and unexposed *D. melanogaster*. Images were taken using confocal microscopy. DmNV *ORF4* was targeted by custom-designed smRNA FISH probes. Single channels are presented in greyscale to facilitate contrast perception. In the merged images, DmNV *ORF4* is represented by yellow and DAPI by cyan. The images presented here are representative of the brains imaged in the investigation. In none of the brains imaged was there any evidence for DmNV *ORF4.*

Brains dissected from *D. melanogaster* infected with DmNV were fixed and analysed with smRNA FISH to target DmNV *ORF4*. None of the brains examined exhibited any signal to indicate presence of DmNV *ORF4*. Meanwhile, nuclei were visualized in all brains. Representative brain images are presented in Figure 3. Over all investigations, at least 20 brains were examined.

### Further characterization of DmNV gut infection by smRNA FISH detection technique

**Figure 4.**
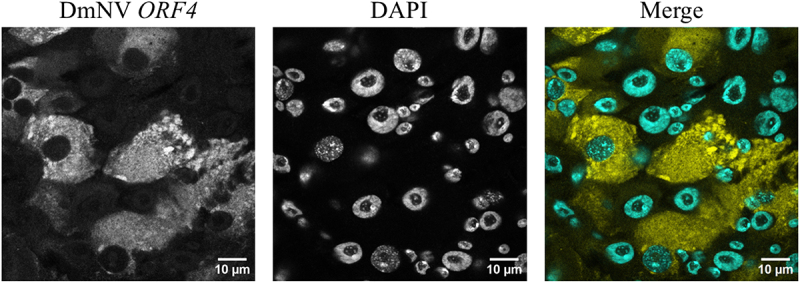
Whole mounted guts from DmNV exposed and unexposed *D. melanogaster*. Images were taken using confocal microscopy. DmNV *ORF4* was targeted by custom-designed smRNA FISH probes. Single channels are presented in greyscale to facilitate contrast perception. In the merged images, DmNV *ORF4* is represented by yellow and DAPI by cyan. Infection appears to be limited to enterocytes, identified by large nuclei. Further, infected enterocytes appear to show an altered pattern of nuclear chromatin condensation, when compared to uninfected nuclei. There is no evidence for DmNV *ORF4* colocalization with DAPI.

**Figure 5.**
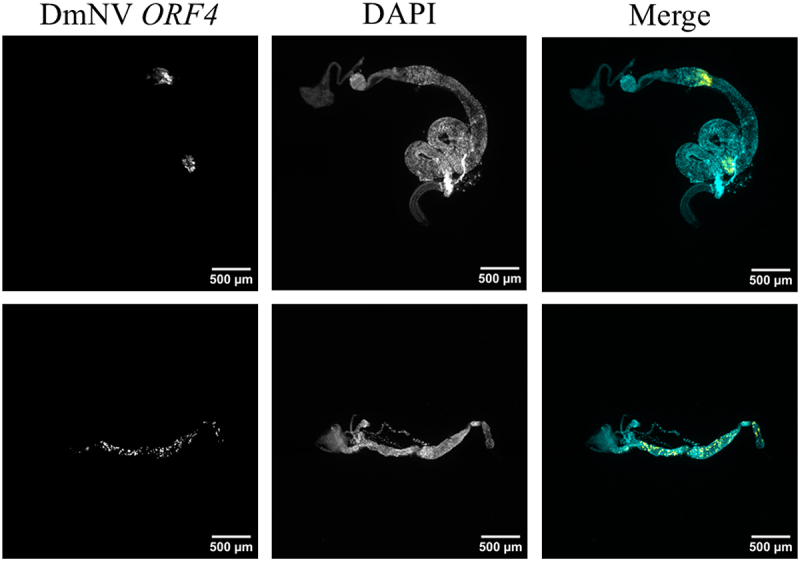
Whole-mounted guts from DmNV infected and uninfected *D. melanogaster*. Images were taken using an inverted fluorescent microscope. DmNV *ORF4* was targeted by custom-designed smRNA FISH probes. Single channels are presented in greyscale to facilitate contrast perception. In the merged images, DmNV *ORF4 is* represented by yellow and DAPI by cyan. The images presented here are representative guts from infected organisms. The top row of panels shows a case where the infection is limited to discrete locales within the midgut, while the bottom row of panels shows a case where the virus invades the gut endothelium following a more diffuse pattern.

While we did not include any staining to aid in identification of specific cell types, the strong signals associated with probes targeting DmNV *ORF4* were largely limited to enterocytes (Figure 4) as identified by their large nuclei [36,37]. We did note a few examples of cells with smaller nuclei, likely intestinal stem cells (ISCs), appearing to be infected, but these were relatively rare occurrences (data not shown). Further, infected enterocytes appear to show an altered pattern of nuclear chromatin condensation when compared to uninfected nuclei. This pattern was unambiguous in the images taken with the 60x oil-immersion objective lens on the confocal microscope (Figure 4). There is no evidence for DmNV *ORF4* colocalization with DAPI. DmNV also exhibits a varied pattern of infection within the *D. melanogaster* gut. In some cases, the infection is limited to discrete locales within the midgut, while in others the virus invades the gut endothelium in a more diffuse pattern (Figure 5).

## Discussion

DmNV and other recently discovered insect Nora viruses appear to represent a newly described ‘picorna-like’ family of viruses with many similarities but key differences to the Picornaviridae family [2–8]. There is still much to learn about these recently discovered insect viruses, and research in this respect is important both in its direct relevancy to insect biology, as well as for virology in general. Further, DmNV is widespread and endemic to both laboratory and wild-caught Drosophila populations [1,38]. Given the relevancy of *D. melanogaster* biology to the fields of genetics and immunology, further characterization of this virus is important.

Heads from DmNV-infected and -uninfected *D. melanogaster* were dissected and RNA extracted from the specimens. From these extractions, a 790 bp product was amplified using primers targeting DmNV *ORF1*. To determine whether the presence of DmNV genetic material within the heads of infected *D. melanogaster* is indicative of direct brain infection, brains were dissected from infected and uninfected *D. melanogaster* and whole mounted for fluorescent microscopy. We initially began our investigation using murine antisera generated against DmNV capsid proteins to probe for signs of brain infection by typical immunohistochemical methods. We did not find any indication of infection in any of the experiments ran (data not shown), as there was no difference in signal between brains dissected from infected or uninfected *D. melanogaster*. Before moving forward with further immunohistochemical trials, we elected to switch approaches for a couple of reasons. First, the antisera we have in our laboratory was generated in a mouse model almost a decade ago and represents a crude serum recovery from mice sensitized with DmNV capsid protein [39] with high potential for off-target binding. Second, during our initial immunofluorescence investigation, we found no evidence for infection within brain tissue, necessitating multiple means of detection to ensure that any lack of evidence we found was not simply due to a problem with our antisera in the application of brain tissue immunohistochemistry. As such, we elected to use an smRNA FISH method to target DmNV-specific nucleic acid sequences to increase the fidelity and specificity of our detection method.

After designing probes and refining a smRNA FISH technique to target DmNV *ORF4* sequences, we dissected brains to look for evidence of infection. We again found no evidence for any signal associated with DmNV-specific nucleic acid within any of the processed brain tissue examined (Figure 3). Meanwhile, we did consistently find DmNV genetic material within the heads of infected *D. melanogaster* via RT-PCR analysis. One reason that DmNV genetic material was detected from dissected heads may be representative of haemolymph transmission of the virus. Previous work shows that DmNV genetic material and capsid protein can be isolated from the haemolymph of infected flies [40]. This could also explain why DmNV-specific siRNA can be found within the heads infected *D. melanogaster* [22], in the context of dicer-2 cleavage products in response to double-stranded replication intermediates. However, the presence of DmNV-specific siRNA does not necessitate DmNV replication within the head. In fact, in *D. melanogaster*, virus-specific siRNA can instead be produced from viral DNA previously reverse transcribed from genomic viral RNA by host machinery [23,24] and carried to uninfected tissue by haemocytes [25]. As such, the DmNV genomic material we identified from dissected heads may simply represent surface contamination of virus-laden faeces, though further investigation is necessary to come to a definitive conclusion.

An investigation by Lopez et al. [41] found several circadian-associated genes, such as *timeless* and *period*, to be upregulated in DmNV-infected *D. melanogaster*, especially later in the course of infection. These genes are of central importance to the biological clock and are expressed in neurons within the *D. melanogaster* brain [42]. This finding could support the hypothesis that DmNV might reach the brains of infected *D. melanogaster*. But in light of the data reported here, perhaps a global immune response in the organism may impact gene expression as opposed to direct viral invasion into brain cells.

In humans, picornavirus infections, such as poliovirus, show central nervous system involvement in only a very small percentage of cases [18]. Indeed, it could be that DmNV does show invasion into the brain in a small subset of cases, explaining why differences in motor function (i.e. geotaxis) are evident between infected and uninfected *D. melanogaster* when the sample size is in the thousands [16]. Another possible reason was the route of infection employed in this study, which was via oral exposure. If the *D. melanogaster* were to be exposed to the virus via injection to establish systemic infection, perhaps oral immune defence mechanisms would be circumvented, allowing the spread to more tissue locales, as has been shown with Drosophila C virus [43].

While the primary implementation of the smRNA FISH method in the present study was not intended to formally characterize infection within the gut, we found evidence of obvious gut infection in roughly 1/3 of the samples tested. This finding is consistent with previous work showing oral introduction of Drosophila C virus to adult iso-*w*^*1118*^ male *D. melanogaster* led to gut infection in only 4 of 20 midguts examined [43]. Further, with DmNV specifically, it has been established that there is a high variation in viral titre between individual *D. melanogaster* from infected stocks, with some of these showing no evidence of infection [36]. Our observations corroborate these data. Additionally, our confocal microscopy images suggest that oral DmNV infection may be confined to enterocytes. These observations are consistent with the results reported by Ekström and Hultmark [36], which found enterocytes to be the main cell type involved in DmNV infection. Franchet et al. [44] recently reported that ISCs also appear to be involved in the pathogenesis of DmNV infection. Similarly, we did note instances of infected cells with smaller nuclei, presumably ISCs. Furthermore, infected enterocytes showed a stark difference in chromatin organization when compared to uninfected enterocytes in the same specimen, which to our knowledge has not been previously described. While the ramifications of this observation are beyond the scope of the present study, further investigation is necessary to validate our assumption. It is important to note that the Witi *Rel*^E23^ stock utilized in the present investigation, while understood to be functionally wild type as a precise excision line not affecting the *Relish* gene, has been shown to harbour relatively high titres of DmNV [1]. Thus, caution must be exercised when generalizing results to other stocks.

Finally, we want to briefly highlight the smRNA FISH detection method that we utilized in this investigation. It proved to be an easy alternative to more traditional immunohistochemical techniques to visualize DmNV within whole-mounted Drosophila tissue. There was negligible noise detected, presumably attributable to the specificity of smRNA FISH in general but also the stringent exclusion criteria we employed for probe sequence dissimilarity to assembled transcripts in the *D. melanogaster* head. When we compared our probes to the entire database of *D. melanogaster* RNA sequences using BLASTn, we did find some sequence similarity, as described in the methods. As such, there is some potential for off-targeting binding; however, the use of dozens of probes and adequate controls helps to control for any background detection and bolsters our results.

The smRNA FISH method of virus detection can also serve as an easy alternative to more technically difficult transgenic methods for visualization of viruses [36,44]. Recently, Hernández-Pelegrín and colleagues [45] used smRNA FISH to visualize RNA viruses in medflies, similar to that described here. The use of RNA FISH in this context represents an exciting era in detection of insect viral infections.

## Supplementary Material

Rokusek et al Supplemental Data file.xlsx

## Data Availability

Data will be made available upon request.
